# Hospital Progress and Finance

**Published:** 1923-08

**Authors:** 


### HOSPITAL PROGRESS AND FINANCE.

### THE LESSON OF THE SEASON.

IN the absence of statistics it is impossible to be certain, but from the information that reaches us from all over the country we are inclined to believe that never have so many fêtes and carnivals been held on behalf of hospitals as in the present year. The number appears overwhelming, and as they are becoming annual affairs, it means that they have come to represent a permanent and regular source of hospital income which no institution can afford to neglect. In the past these events were supplementary. To-day they are a principal means of revenue, and the element of charity is supplied more by the hard work given in their organisation than in the money paid at the gates. An immense amount of energy is freely expended, and thus the suggestion sometimes made that charity has become a business is happily an exaggeration. The mass of people never did subscribe to hospitals. They were supported first by the generous few, then by sections of the community organised among themselves as annual donors, and now that these resources are insufficient the general public is being provided with amusement in an endless variety of ways, which, through the generosity of the organisers and their friends, leave a handsome profit for charity. On the surface the change seems immense, but what has really happened is rather that the support of the mass of the population is now essential and can only be gained in the indirect way provided by a fête. The few continue as before to give directly if they have the means, or by personal service if they have not, and personal service is now largely expressed by the volunteer work of organisation. Even appeals now largely rely less on propaganda than on organised amusement, of which the direct collection of money is only a side-show. The general appeal is an appeal to the instinct for pleasure. The generosity appears in providing it, and the means for it, without reward. From this point of view it may even be said that more people are giving to hospitals than ever, though annual service is taking the place of the annual subscription.

### THE DEVELOPMENT OF AFTER-CARE.

THE auxiliary care of hospital patients has been made the subject of inquiry by the Liverpool Council of Voluntary Aid. The objects in view are to relieve patients from domestic anxiety when in hospital and to provide for their after care and assistance on discharge. The existing means for these ends are described, and a plea is made for somebody to do for adults that which the Child Welfare Association does for children. At present, it is pointed out, only certain forms of auxiliary care, such as convalescent treatment and medical appliances, are provided. A Personal Service Society already exists, and its difficulty in supplying special nourishment to ex-patients has given rise to the inquiry. The society is willing to co-operate with the hospitals' almoners in undertaking auxiliary care. The cost would be £300 a year, which, it is thought, the Saturday Fund or the Red Cross might provide. Besides this, the society might help to collect from ex-patients the voluntary gifts they often are ready to make, but which lapse at present because they fail to go regularly to the hospitals to make them. As it is, the almoners have no time to follow up these ex-patients in their homes. It will be seen from this inquiry that much is already done, but that after-care knows no defined limits and may be needed at a point beyond which the hospitals unaided cannot go.

### RECONSTRUCTION AT THE WESTMINSTER.

THE closing of the Westminster Hospital at the beginning of July for repairs and alterations will keep the building empty of patients until next January. In both in- and out-patients departments alterations are to be made. These include a new storey at the west end of the hospital, a new operating theatre and larger accommodation for casualties and out-patients. The estimated cost is £45,000, of which nearly half is already in hand, and for the rest a public appeal will be made in October. The staff meantime will benefit in various ways. Sixteen sisters are on holiday with full pay and ward allowances, and the probationers have been distributed among other hospitals so as to continue their training. The administrative staff will be kept busy, and the reconstruction scheme presumably proves that all idea of moving the hospital is at an end. In this respect the occasion marks an important date in the hospital's history.

### THE PRINCE AND EAST DENBIGHSHIRE.

NOW that the building of the new hospital at Wrexham has begun, an ambitious scheme may be said to be approaching realisation. In 1918 it was resolved to build a hospital as a War memorial to the men of Wrexham and East Denbighshire; the old infirmary, built in 1833, provides only forty beds. The new hospital, which will cost £70,000, will accommodate 100, and the Prince of Wales has promised to lay the foundation-stone on October 12. By that date it is hoped to raise the £11,000 still required toward the cost, a relatively small sum thanks to the generosity of the industrial workers of the county who, by contributing 2d. a week for three years have subscribed £18,564, and to a munificent grant of £30,000 from the William and John Jones Hospital Trustees. By the end of next year the hospital should be finished and equipped, and the number of beds adequate at least to the immediate needs of the community. If the colliery owners and employers are not behind their men in generosity the new hospital could be opened free of debt. A War memorial hospital that is laggardly supported does little honour to the memories for which it was raised.

### THE WOMAN QUESTION AT NOTTINGHAM.

THE question whether women should serve on the monthly board of the Nottingham General Hospital has been the subject of a special meeting of governors. In 1909 the proposal was negatived, and since then it has become increasingly discussed. Finally, by 93 votes to 80, it was decided to admit women; and thus the controversy is closed. The voting shows that opinion was fairly equally divided, and that some felt keenly on the matter may be judged from a letter which the Chairman, Mr. F. Acton, read at the close. The writer, a gentleman, declared that if the vote went against the admission of women, he would subscribe one hundred guineas to the hospital and increase his annual subscription by five guineas a year! Such a die-hard deserves to be remembered, but the only consolation we can offer is that the Nottingham Hospital is now doing what has become general, and, so far as we know, has led to no regrets. If any member of a hospital board, of either sex, in which women are included has experiences to the contrary, we shall be glad to publish them. But our belief is that the question is happily settled.

---

### RECONSTRUCTION AT BRIGHTON.

MR. HARRY PRESTON, President of the Royal Sussex County Hospital, Brighton, in the endeavour to set its finances straight again, has issued an appeal for a thousand annual subscribers of one guinea. This valuable form of income is always hard to secure, but is especially desirable at Brighton, where such a nucleus is needed after the recent financial troubles, inquiry and criticism. In all this, however, the true friends of the hospital have an opportunity that may not come again to prove their loyalty and to set an example which has never been more needed. Beside this, the new orthopædic department will be finished in September so that the reconstruction of the hospital's activities in all directions is being taken in hand. In March, sixty-five of the closed beds were reopened, and if Mr. Preston's lead is followed the remainder should be opened next month. A difficult corner is being turned, and Mr. Preston's personal example, in activity and generosity alike, deserves a response even greater than that which he has immediately asked for.

---

### THE ABERDEEN HOSPITALS' SCHEME.

A CRITICAL moment has been reached in the hospital problem at Aberdeen, where the long-considered Joint Hospital scheme is about to be definitely adopted or rejected. The Hospital for Sick Children can no longer delay its own projects of expansion, and therefore the ambitious plan to centralise the hospitals on the Foresterhill site, in which the concurrence of the Town Council is important, has been referred by it to a special committee, whose decision is expectantly awaited. Great importance attaches to the verdict, and despite difficulties or objections, many hopes centre on the joint scheme which promises to Aberdeen the realisation of what has been popularly called a hospital city, in which the needs of the Royal Infirmary, the Royal Hospital for Sick Children, the University's Medical and Scientific Departments, together with a tuberculosis hospital, would be met.

### ST. LUKE'S AND THE MIDDLESEX.

THE Middlesex Hospital has taken another step in the treatment of nerve cases by opening two wards, in co-operation with St. Luke's Hospital, for cases of functional nervous disorder which require in-patient hospital treatment. Hitherto no beds were available for such sufferers, nor have a general and a special hospital combined their resources. The patients chosen, it is explained, are not mental deficients nor likely to cause annoyance to others, but rather borderline cases for whom, till very recent times, no provision has been made. The Middlesex has made several co-operative experiments since the war, and this latest example will be watched with interest.

---

### THE HOSPITAL MARKET.

THE prospective sale of the East Ham Cottage Hospital is still undecided, though the Town Council and the hospital committee have been negotiating for more than a year. A memorial hospital was proposed and money has been collected, but the hospital committee will not commit itself further until it knows whether the Town Council will really purchase the present building. A sum of £15,000 is nominally the purchase price that the Council tentatively considered, but reflection has apparently made the Council think with other people that the figure is excessive. The Council wants, or wanted, the Cottage Hospital for a maternity home, and then as a common centre for its clinics. At the moment, a new committee is investigating the matter, and seeing how many different things depend upon a decision, we hope that some decision will soon be made. The position is becoming ludicrous, and the Council must really discover what mind it has to make up.

---

### AN INTERESTING DISCOVERY.

AN interesting discovery has been made at the Poplar Hospital for Accidents where, during some excavations undertaken to strengthen the foundations of the building, the workmen found a collection of bones. These were examined by Professor Sir Arthur Keith, curator of the Museum of the Royal College of Surgeons, and it is thought that they may belong to an animal of the Bronze Age. The bones were discovered at a depth of seventeen feet on a gravel bed below five feet of silt; and, whatever their origin, incidentally draw attention to a work of capital expenditure which should now have an unlooked-for claim upon the interest and support of the public. It is a curious and remarkable find which Mr. D. H. Lindsay, the hospital secretary, will know how to use to the best advantage.

---

### WIRELESS IN HOSPITALS.

THE installation of wireless apparatus is being considered at several hospitals. One children's hospital possesses one, and even cottage hospitals are hoping to follow suit.

